# Computing the sequence of *k*-cardinality assignments

**DOI:** 10.1007/s10878-022-00889-4

**Published:** 2022-07-24

**Authors:** Amnon Rosenmann

**Affiliations:** Institute of Discrete Mathematics, Graz University of Technology, Steyrergasse 30, 8010 Graz, Austria

**Keywords:** *k*-cardinality assignment problem, Parametric assignment algorithm, Max-plus algebra, Full characteristic maxpolynomial, 90C10, 15A80, 15A15

## Abstract

The *k*-cardinality assignment (*k*-assignment, for short) problem asks for finding a minimal (maximal) weight of a matching of cardinality *k* in a weighted bipartite graph $K_{n,n}$, $k \le n$. Here we are interested in computing the sequence of all *k*-assignments, $k=1,\ldots ,n$. By applying the algorithm of Gassner and Klinz (2010) for the parametric assignment problem one can compute in time ${\mathcal {O}}(n^3)$ the set of *k*-assignments for those integers $k \le n$ which refer to essential terms of the full characteristic maxpolynomial ${\bar{\chi }}_{W}(x)$ of the corresponding max-plus weight matrix *W*. We show that ${\bar{\chi }}_{W}(x)$ is in full canonical form, which implies that the remaining *k*-assignments refer to semi-essential terms of ${\bar{\chi }}_{W}(x)$. This property enables us to efficiently compute in time ${\mathcal {O}}(n^2)$ all the remaining *k*-assignments out of the already computed essential *k*-assignments. It follows that time complexity for computing the sequence of all *k*-cardinality assignments is ${\mathcal {O}}(n^3)$, which is the best known time for this problem.

## Introduction

A fundamental problem in combinatorial optimization is the linear assignment problem, which is special type of a minimum cost flow problem, or even more generally, of a linear programming problem. It is common to describe it in terms of “agents” and “tasks”. The tasks are to be performed by the agents in such a way that each agent is assigned to at most one task and no task may be performed by more than one agent. For each agent-task pair a “cost” is attached, telling how much does it cost for the specific agent to perform the specific task. The assignment problem asks for finding an optimal assignment of agents to tasks such that the total cost is minimal. Clearly, an analogous problem asks for finding an assignment which is optimal with regard to a maximal total cost.

The assignment problem has numerous and diverse practical applications. Some examples: assigning workers to jobs, e.g. salespersons to sales territories, taxi drivers to different locations, assigning machines to factory orders, computer programs to processors, teachers to classes, contracts to bidders, switching satellites time slots assigning transmitting to receiving earth stations, sensors to airplane tracks in air traffic control.

In its basic form the number of agents equals the number of tasks (a “balanced” assignment). Here we deal with a more general problem. Given *n* agents and *n* tasks and a number $k \le n$, the problem is to find an optimal matching of *k* agents to *k* tasks such that the total cost of this assignment is minimal among all assignments of cardinality *k*. In practice, the assignment problem may repeat itself many times, where the number of tasks in the single assignments alters between 1 and *n*. In this case we are interested in computing in advance the optimal cost of all *k*-cardinality assignments (*k*-assignments, for short), for *k* running from 1 to *n*, in an efficient way that outperforms the total cost of computing each of the *k*-assignments separately. This last problem is the topic of the current paper.

The assignment problem can naturally be represented as a weighted bipartite graph, where one set of vertices represents the agents and the other represents the tasks, and the weights on the edges represent the corresponding costs.

Among the generalizations of the standard assignment problem is the parametric assignment problem, which is a special case of a parametric linear programming problem (Murty 1983). In the parametric assignment problem the costs are not restricted to be constants but functions of a parameter $\lambda$. When the parametric costs are linear functions of $\lambda$ then the optimal cost of an *n*-assignment forms a piecewise linear function of $\lambda$ defined over the real numbers and with finitely-many pieces (intervals). The goal is to find optimal assignments at each of these intervals in an incremental (or successive) and efficient way. A special and efficient algorithm of Gassner and Klinz (G-K) (2010) for the parametric linear assignment problem, in which the weight (cost) at each edge $e_{ij}$ is either $w_{ij}$ or $\lambda + w_{ij}$, does exactly that and its time complexity is ${\mathcal {O}}(n^3)$.

The question is, how do we obtain the optimal *k*-assignments by solving the parametric assignment problem? The G-K algorithm works also when the cost function of $e_{ij}$ is $\min \{\lambda , w_{ij}\}$. Suppose now that we assign to *each* edge $e_{ij}$ the weight $\min \{\lambda , w_{ij}\}$ and solve the parametric assignment problem with the G-K algorithm. Then, if within some interval we obtain an optimal assignment in which $n-k$ of the edges have the value $\min \{\lambda , w_{ij}\}=\lambda$ and *k* edges have the value $\min \{\lambda , w_{ij}\}=w_{ij}$ then the latter form an optimal *k*-assignment for that specific *k*. It follows that after completing the algorithm we obtain a list of optimal *k*-assignments in time ${\mathcal {O}}(n^3)$. This is better than the total complexity of ${\mathcal {O}}(n^4)$ achieved by computing separately for each *k* the optimal *k*-assignment since the best time complexity for solving a single *k*-assignment problem is also ${\mathcal {O}}(n^3)$ (which is quite surprising, given that the optimal *k*-assignment is chosen among $\frac{[n(n-1)\cdots (n-k+1)]^2}{k!}$ possibilities).

The problem is that, in general, the above procedure will not produce $$all $$ the *k*-assignments, for $$k = 1, \ldots , n$$. Here comes our contribution by showing how to obtain in a simple way the remaining *k*-assignments in an overall time of $${\mathcal {O}}(n^2)$$. It follows that the *entire* sequence of *k*-assignments can be computed in time $${\mathcal {O}}(n^3)$$, which is the best time complexity that can be achieved with the known algorithms.

The idea is to exploit the special case of the parametric assignment problem in which the parameter $\lambda$ appears at all the edges $e_{ij}$ of the bipartite graph as a cost function $\min \{\lambda , w_{ij}\}$. For convenience, we solve here the *maximal* assignment problem (clearly, when the costs are set to be $-w_{ij}$ then maximal assignment gives the minimal assignment with the original costs $w_{ij}$). The natural setting for working with the maximum operation is max-plus algebra, where the operations of maximum and addition replace the standard addition and multiplication operations, respectively. Given an $n \times n$ matrix *A* with entries $a_{ij} = w_{ij}$, its “full characteristic maxpolynomial” ${\bar{\chi }}_{A}(\lambda )$ is defined to be the max-permanent of the matrix with entries $\max \{\lambda , w_{ij}\}$. Then the coefficients of the powers $\lambda ^{n-k}$ of ${\bar{\chi }}_{A}(\lambda )$ are exactly the (maximal) values of the *k*-assignments. Moreover, unlike the case with the characteristic maxpolynomial $\chi _A(\lambda )$, where the parameter appears on the diagonal of the matrix, we show that the *full* characteristic maxpolynomial ${\bar{\chi }}_{A}(\lambda )$ is in “full canonical form”, and this property allows us to compute efficiently the *k*-assignments that remain after applying the G-K algorithm. That is, we show that the remaining *k*-assignments can be extracted out of those that were already computed by the G-K algorithm and there is no need to consider the set of all costs $w_{ij}$.

### Related work

A well-known algorithm that solves the assignment problem is the “Hungarian algorithm” (or “Hungarian method”) of Kuhn (1955, 1956), based on the works of König (1916) and Egerváry (1931). Munkres (1957) showed that the algorithm is of strongly polynomial time complexity, in fact ${\mathcal {O}}(n^4)$. Later Edmonds and Karp (1972) and Tomizawa (1971) gave a version of the algorithm whose running time is ${\mathcal {O}}(n^3)$.

The best known time complexity for solving the *k*-cardinality assignment problem for a specific $k \le n$ in a weighted full bipartite graph $K_{n,n}$ is also ${\mathcal {O}}(n^3)$, for example through network flow. In fact, the algorithm that is based on the successive shortest path algorithm for the minimum flow problem, which augments one unit of flow at each iteration (see Ahuja et al. 1993, Ch. 9.7 and Ch. 12.4), computes the sequence of *k*-assignments, for $k=1,\ldots ,n$, in time ${\mathcal {O}}(n^3)$.

The algorithm of Gassner and Klinz (G-K) (2010) for the parametric assignment problem is of time complexity ${\mathcal {O}}(n^3)$. It is based on the algorithm of Young et al. (1991) for the parametric shortest path problem. In Young et al. (1991) the authors make use of the Fibonacci heap data structure of Fredman and Tarjan (1987) to improve the time complexity of the algorithm of Karp and Orlin (1981) for the parametric shortest path from ${\mathcal {O}}(n^3\log n)$ to ${\mathcal {O}}(n^3)$. At each iteration of the G-K algorithm a new “breakpoint” is computed and the shortest path tree in the “residual graph” is updated. At some point a cycle emerges that forces the tree to be ill-defined and then the next parametric assignment is computed. We remark that, in general, known algorithms for the parametric linear *programming* problem may have exponential number of breakpoints (Carstensen 1983). In fact, integer linear programming is an NP-complete problem even when the unknowns are binary.

A generalization of the G-K algorithm to maxpolynomials of any degree (not just linear ones) was introduced by Hook (2016).

In Gassner and Klinz (2010) applied their algorithm to efficiently compute the characteristic maxpolynomial of a matrix. As with the standard characteristic polynomial, the parameter (or variable) appears on the diagonal of the matrix. The coefficients of the characteristic maxpolynomial are the (optimal) “principal” *k*-assignments, that is, referring to principal square submatrices. However, the G-K algorithm computes the assignments that refer to “essential” terms and possibly some of the “semi-essential” terms of the characteristic maxpolynomial, and not the “inessential” terms and hence, in general, not all the principal *k*-assignments are computed (and to the best of our knowledge, it is not even known if a polynomial-time algorithm exists for computing these *k*-assignments).

Here we compute the *full* characteristic maxpolynomial, where the parameter appears on all entries of the matrix, thus the coefficients of this maxpolynomial are the standard *k*-assignments, since they are optimal among all $k \times k$ submatrices. Moreover, in the full characteristic maxpolynomial there are no inessential terms, only essential and semi-essential ones. This follows from the fact that the full characteristic maxpolynomial is in full canonical form, for which we give here a simple proof (for another proof, based on the fact that the matrix of the corresponding linear programming problem is totally unimodular, see Rosenmann et al. 2019). The fact that there are no inessential terms allows us to compute in an efficient way the *k*-assignments that were left after applying the G-K algorithm.

### Organization of the article

In Sect. 2 we define the *k*-linear assignment problem and represent it as a matching problem in a weighted bipartite graph and as a linear programming problem.

In Sect. 3 we introduce briefly the Max-plus algebra notions that are relevant to our problem and we show that the full characteristic maxpolynomial is in full canonical form (FCF). Then, in Sect. 4 we show that the sequence of *k*-assignments is concave, thus providing another proof for the FCF property of the full characteristic maxpolynomial.

In Sect. 5 the computation of the *k*-assignments is presented. First, we describe the G-K algorithm for the parametric assignment problem when applied in the max-plus setting to the matrix with a parameter in all its entries, which is relevant for computing the *k*-assignments. Then we present our algorithm for computing the *k*-assignments that remain to be computed after completing the G-K algorithm. We conclude with a time complexity analysis.

## The *k*-cardinality linear assignment problem

We are given a complete bipartite graph (biclique) $K_{n,n} = G(U,V;\!\!E)$ consisting of two disjoint sets of vertices $U = \{u_1, \ldots , u_n\}$ and $V = \{v_1, \ldots , v_n\}$, and an edge set *E*, adjoining each vertex $u_i \in U$ with each vertex $v_j \in V$ by an edge $e_{ij}$. In addition, we are given a weight (cost) function  which assigns each edge $e_{ij}$ a weight $w_{ij}$. The *k*-cardinality Linear Assignment Problem, or *k*-LAP (see e.g. Dell’Amico and Martello 1997; Dell’Amico et al. 2001; Volgenant 2004; Bai 2009; Belik and Jörnsten 2016; Bhunia et al. 2017; Kumar and Purusotham 2018; Prakash et al. 2022), asks for finding an optimal (here optimal is maximal) weight of a matching (independent edge set) of cardinality *k*, where $k \le n$. That is, we are looking for a set $M_k \subseteq E$ of *k* pairwise non-adjacent edges in $K_{n,n}$ that is of maximal total weight:

2.1 $$\begin{aligned} \max _{\{i_1,\ldots ,{i_k}\},\{j_1,\ldots ,{j_k}\} \subseteq [1..n]} \; \Sigma _{l=1}^k w_{i_l j_l}, \end{aligned}$$

where both $\{i_1,\ldots ,{i_k}\}$ and $\{j_1,\ldots ,{j_k}\}$ consist of distinct indices.

### Remarks 2.1

1. Here we compute the maximal assignments. For computing the minimal assignments we can either first multiply the weights by $-1$ and then compute the maximum, or compute directly the minimum in the analogous setting of .
2. If the problem refers to sets *U* and *V* of different cardinalities *n* and $m < n$, respectively, then we can always introduce $n-m$ spurious (dummy) vertices and $n(n-m)$ spurious edges of weights $-\infty$ ($+\infty$ in the minimal assignment case) so that *U* and *V* become of equal cardinality.
3. We refer to Burkard et al. (2012) for a comprehensive treatment of linear and related assignment problems.

Representing the weight function as an $n \times n$ matrix $W = (w_{ij})$ over , the *k*-LAP is about finding *k* elements of *k* different rows and *k* different columns, such that their sum is maximal. It can be formulated as an Integer Programming (IP) problem over the variables $x_{i,j}$, for $i,j = 1,\ldots ,n$, as follows:

2.2 $$\begin{aligned} \text{ maximize } \, \sum _{i=1}^n \sum _{j=1}^n w_{ij} x_{ij} \end{aligned}$$

subject to the conditions

2.3 $$\begin{aligned}&\sum _{j=1}^n x_{ij} \le 1 \qquad (i = 1, \ldots , n), \nonumber \\&\sum _{i=1}^n x_{ij} \le 1 \qquad (j = 1, \ldots , n), \nonumber \\&\sum _{i=1}^n \sum _{j=1}^n x_{ij} = k, \nonumber \\&x_{ij} \in \{0,1\} \qquad (i,j = 1, \ldots , n). \end{aligned}$$

If we replace the last condition $x_{ij} \in \{0,1\}$ in (2.3) by the inequalities

2.4 $$\begin{aligned} x_{ij} \ge 0 \qquad (i,j = 1, \ldots , n), \end{aligned}$$

we obtain the corresponding Linear Programming (LP) problem.

A LAP can also be described in the language of network flows or in terms of matroids. In what follows we use the term *k*-assignment when referring to *k*-cardinality linear (optimal) assignment.

## Max-plus algebra

The values of the *k*-assignment problem, $k=1, \ldots , n$, appear as the coefficients of the full characteristic polynomial of the weight matrix *W* in the setting of max-plus algebra. In this section we present the relevant background about max-plus algebra.

Max-plus algebra (Butkovič 2010) is an algebra over the semifield , equipped with the operations of addition $a\oplus b$ defined as $\max (a,b)$ and multiplication $a \odot b$ defined as $a+b$ in standard arithmetic, with the unit elements $-\infty$ for addition and 0 for multiplication. For the sake of readability, we suppress the multiplication sign $\odot$, writing *ab* instead of $a \odot b$ and $ax^3$ instead of $a \odot x^{\odot 3}$. Also, when an indeterminate *x* appears without a coefficient, as in $x^n$, then its coefficient is naturally the multiplicative identity element, i.e. 0.

A **formal maxpolynomial** of degree *n* over  in the indeterminate *x* is an expression of the form

3.1 $$\begin{aligned} p (x)= \bigoplus _{k=0} ^n a_k x^k = \max \{a_k + k x: k=0, 1, \ldots , n \} \end{aligned}$$

with . Each element of the generated algebra under the max and plus operations can be reduced to a unique expression of the form (3.1) with *x* treated as an indeterminate.

Given a formal maxpolynomial *p*(*x*), the corresponding maxpolynomial *function*  is convex and piecewise-affine. Unlike the situation in standard arithmetic, two distinct formal maxpolynomials $p_1(x)$ and $p_2(x)$ may represent the same maxpolynomial function, that is, for each *x*, the values of ${\hat{p}}_1(x)$ and ${\hat{p}}_2(x)$ in  are the same. For suppose that a maxmonomial $a_kx^k$ of *p*(*x*) has the property that for every value of *x* there exists another maxmonomial $a_lx^l$ of *p*(*x*), $l \ne k$, such that $a_kx^k \le a_lx^l$ (or, in standard arithmetic, $\max \{a_k + k x < a_l + l x$), then $a_kx^k$ does not contribute to the function ${\hat{p}}(x)$ and thus may be omitted or replaced by another maxmonomial $a'_kx^k$ (at least when $a_k' < a_k$). The term $a_kx^k$ is said to be an **essential** term of *p*(*x*) when ${\hat{p}}(x) = a_kx^k$ (as functions) in some interval of , . When $a_kx^k < {\hat{p}}(x)$ for all values of *x* then $a_kx^k$ is said to be **inessential**. Otherwise, when ${\hat{p}}(x) = a_kx^k$ at a single point, then we say that $a_kx^k$ is **semi-essential** (in the literature there is no distinction between an inessential and a semi-essential term).

For each function ${\hat{q}}(x)$ there exists a unique maxpolynomial representation *p*(*x*), such that every power $x^k$ of *p*(*x*) appears with the maximal possible coefficient $a_k$, as long as the functional equality ${\hat{p}}(x)={\hat{q}}(x)$ holds. We then say that *p*(*x*) is in **full canonical form** (in FCF) (Cuninghame-Green and Meijer 1980). It follows that when *p*(*x*) is in FCF then all its maxmonomials are either essential or semi-essential.

The **max-plus roots** (**tropical** **roots**) $\lambda \ne -\infty$ of a maxpolynomial *p*(*x*) are the points at which ${\hat{p}}(x)$ is non-differentiable. The multiplicity of a root equals the change of the slope of ${\hat{p}}(x)$ at that root. Equivalently, the roots of *p*(*x*) are the values $\lambda \ne -\infty$ of *x* at which at least two maxmonomials $a_kx^k, a_{k+1}x^{k+1}, \ldots , a_{k+d}x^{k+d}$ in the corresponding full canonical form satisfy $a_k \lambda ^k = a_{k+1}\lambda ^{k+1} = \cdots = a_{k+d}\lambda ^{k+d} = {\hat{p}}(\lambda )$ and the multiplicity of the root $\lambda$ is then *d*, the difference between the largest and the smallest index of these maxmonomials. We also count $-\infty$ as a root with multiplicity *d* whenever $a_0, a_1, \ldots , a_{d-1}$ are all equal to $-\infty$ and $a_d \ne -\infty$.

### Proposition 3.1

(see Rosenmann et al. 2019) Let $p(x) = \bigoplus _{k=0}^{n} a_k x^k$ be a maxpolynomial of degree $n \ge 1$ and let $\lambda _1 \le \lambda _2 \le \cdots \le \lambda _n$ be its roots. Then the following are equivalent characterizations of *p*(*x*) to be in full canonical form.

1. $$p(x) = a_n (x \oplus \lambda _1) \cdots (x \oplus \lambda _n)$$ formally.
2. $$\lambda _k = a_{k-1} - a_k$$, $$k = 1, \ldots , n$$ (here $$-\infty - (-\infty ) = -\infty $$).
3. Concavity: $$a_k \ge (a_{k-1}+a_{k+1})/2$$, $$k = 1, \ldots , n-1$$.
4. ${\hat{p}}(\lambda _k) = a_k \lambda _k^k$ ($a_k + k \lambda _k$ in standard arithmetic), $k = 1, \ldots , n$.

### The (full) characteristic maxpolynomial of a matrix

Given an $n \times n$ matrix , the **max-plus permanent** of *A* is

$$\begin{aligned} {{\,\mathrm{\mathrm {maxperm}}\,}}(A) = \bigoplus _{\sigma \in {\mathfrak {S}}_n} a_{1 \sigma (1)} \cdots a_{n \sigma (n)}, \end{aligned}$$

where ${\mathfrak {S}}_n$ is the group of permutations on $[n] =\{1, \ldots ,n \}$. The **characteristic maxpolynomial** of *A*, defined in Cuninghame-Green (1983), is

$$\begin{aligned} \chi _A (x) = {{\,\mathrm{\mathrm {maxperm}}\,}}(x I \oplus A), \end{aligned}$$

where *I* is the max-plus identity matrix with 0 on the main diagonal and $-\infty$ off-diagonal. It is a maxpolynomial of degree *n*, say $\chi _A(x)=\bigoplus _{k=0}^n a_k x^{n-k}$, with $a_k$ being the maximal value among all principal maxpermanent minors of order *k* of *A*. The (tropical) roots of the characteristic maxpolynomial $\chi _A (x)$ are called the **max-plus eigenvalues** of *A*. As shown in Akian et al. (2001), they can be asymptotically computed from the eigenvalues of an associated parametrized standard matrix with exponential entries.

Another maxpolynomial related to *A* is its **full characteristic maxpolynomial**, defined as

$$\begin{aligned} {\bar{\chi }}_{A} (x) = {{\,\mathrm{\mathrm {maxperm}}\,}}(x {{\textbf {0}}} \oplus A), \end{aligned}$$

where ${{\textbf {0}}}$ is the matrix with 0 in all its entries (see Hook 2015). The difference between ${\bar{\chi }}_{A} (x)$ and $\chi _A (x)$ is that in ${\bar{\chi }}_{A} (x)$ the indeterminate *x* appears in all entries instead of just on the diagonal. The coefficients $a_k$ of ${\bar{\chi }}_{A}(x)=\bigoplus _{k=0}^n a_k x^{n-k}$ equal the maximal value among all maxpermanent minors (not just principal maxpermanent minors) of order *k* of *A*. That is, $a_k$ equals the value of a *k*-assignment with weight matrix *A*. The (tropical) roots of the full characteristic maxpolynomial ${\bar{\chi }}_{A} (x)$ are called the **max-plus singular values** of *A* (Hook 2015). Similar to the max-plus eigenvalues, the max-plus singular values can be asymptotically computed from standard singular values (see Hook 2015; De Schutter and De Moor 2002).

An essential (formal) property of the full characteristic maxpolynomial, which distinguishes it from the characteristic maxpolynomial, is that it is in full canonical form. This property allows us to compute efficiently all the *k*-assignments.

#### Proposition 3.2

(see e.g. Rosenmann et al. 2019) Let . Then the full characteristic maxpolynomial ${\bar{\chi }}_{A} (x)$ is in FCF.

Indeed, the coefficients of ${\bar{\chi }}_{A}(x)=\bigoplus _{k=0}^n a_k x^{n-k}$ are the solutions of the IP problem (2.2), (2.3). If we look at the corresponding LP problem (2.2) under the conditions (2.3), with (2.4) replacing the last condition of (2.3), then it is clear that the set of solutions $w_k$ to the *k*-LP problems form a concave set: $w_k \ge (w_{k-1}+w_{k+1})/2$, which is one of the equivalent conditions appearing in Proposition 3.1. The proof would be complete if these solutions were integers. However, since the standard form of the constraint matrix of our LP problem is a $(2n+1)\times (n^2+2n)$ *totally unimodular* matrix (Dell’Amico and Martello 1997) then the solutions are indeed integers: $w_k = a_k$.

## Concavity of the sequence of *k*-assignments

We give here a direct proof of the FCF property of the full characteristic polynomial without referring to linear programming or network flow but rather demonstrate it on the bipartite graph. The method of proof will serve us in what will follow.

First, we show the known fact (see Dell’Amico and Martello 1997) that it is always possible to form a (maximal) $(k+1)$-assignment from the set of vertices matched by a *k*-assignment and an additional pair of vertices. Given a set $E'$ of matched pairs in a bipartite graph $G(U,V;\!\!E)$, an **alternating path** in *E* with respect to $E'$ is a path of positive length that alternately switches between edges of $E'$ and those of $E {\setminus } E'$. If an alternating path starts and ends in a vertex of $E {\setminus } E'$ then it is an **augmenting path** (may also be of length 1).

### Lemma 4.1

Let $K_{n,n} = G(U,V;\!\!E)$ be a complete bipartite weighted graph on vertices $U=\{u_1,\ldots ,u_n\}$ and $V=\{v_1,\ldots ,v_n\}$. Let $M_k$, $k=1,\ldots ,n$ be a sequence of *k*-assignments on $K_{n,n}$. Then, after possibly renaming the vertices, the set of matched vertices in $M_k$ may be chosen to be $\{u_1,\ldots ,u_k, v_1,\ldots ,v_k\}$, for $k=1,\ldots ,n$.

### Proof

Let $E_k, E_{k+1}$ be the set of edges of $M_k, M_{k+1}$, respectively. The edges of $E_k \cup E_{k+1}$ form a disjoint (with no common vertices) union of alternating paths. Because $\vert E_{k+1}\vert > \vert E_k \vert$, at least one of these paths *p* is augmenting: it starts in a vertex $u_{k+1} \in M_{k+1} {\setminus } M_k$, ends in a vertex $v_{k+1} \in M_{k+1} {\setminus } M_k$, and all the inner vertices are in $M_k \cap M_{k+1}$. By the maximality of total weight of the two assignments, we may take the rest of the matches of $M_k$ to be also in $M_{k+1}$, that is, $M_{k+1} = M_k \triangle p$, the symmetric difference of $M_k$ and *p*. Hence, $M_{k+1}$ contains exactly one pair of vertices that is not in $M_k$. $\square$

In terms of matrices, Lemma 4.1 says that given a matrix , there exist permutation matrices , such that the *k*-assignments in $W'=PWQ$ occur on its leading principal $k \times k$ submatrices, for $k=1,\ldots ,n$.

We give now an alternative Proof of Proposition 3.2 in the language of *k*-assignments on bipartite graphs. We define a 0-assignment to be $M_0 = \emptyset$ with weight $w_0=-\infty$.

### Proposition 4.2

Given a complete bipartite weighted graph $K_{n,n}$, $n \ge 2$, the sequence of weights $w_k$, $k=0,\ldots ,n$, of its *k*-assignments is concave.

### Proof

Let $M_{k-1}$, $M_k$ and $M_{k+1}$ be 3 consecutive assignments with weights $w_{k-1}$, $w_k$ and $w_{k+1}$, respectively. We need to show that $w_k \ge (w_{k-1}+w_{k+1})/2$. By Lemma 4.1, we may assume that $M_{k+1}$ matches the vertices matched in $M_{k-1}$ and additional 4 vertices and it consists of some of the pairs matched in $M_{k-1}$ and other matched pairs which form two disjoint maximal augmenting paths $p_1,p_2$ with the rest of the edges of $M_{k-1}$. That is,

$$\begin{aligned} p_1 \cap p_2 = \emptyset \end{aligned}$$

and

$$\begin{aligned} p_1 \cup p_2 = M_{k+1} \triangle M_{k-1}. \end{aligned}$$

For $$i=1,2$$, let

$$\begin{aligned} g_i = w(p_i \cap M_{k+1}) - w(p_i \cap M_{k-1}) \end{aligned}$$

be the gain exhibited by $p_i$, that is, the difference between the total weight of the matches of $M_{k+1}$ and those of $M_{k-1}$ in $p_i$. Then

$$\begin{aligned} w_{k+1} = w_{k-1} + g_1 + g_2. \end{aligned}$$

Suppose, without loss of generality, that $g_1 \ge g_2$. Then we form a (not necessarily maximal) matching $M'_k$,

$$\begin{aligned} M'_k = M_{k-1} \triangle p_1 \end{aligned}$$

and we have

$$\begin{aligned} g_1 = w(M'_k) - w_{k-1}. \end{aligned}$$

By the maximality of $w_k$,

$$\begin{aligned} w_k \ge w(M'_k) = w_{k-1}+g_1 = \frac{2w_{k-1}+2g_1}{2} \ge \frac{2w_{k-1}+g_1+g_2}{2} = \frac{w_{k-1}+w_{k+1}}{2} \end{aligned}$$

and the proof is complete. $\square$

### Example 4.3

Let

$$\begin{aligned} W = \begin{bmatrix} -\infty &{} 8 &{} 5 &{} 0 \\ 10 &{} 8 &{} 5 &{} -\infty \\ 8 &{} 0 &{} 5 &{} 4 \\ 5 &{} 4 &{} -\infty &{} -\infty \end{bmatrix} \end{aligned}$$

be the matrix representation of a weighted bipartite graph $K_{4,4}$ with disjoint sets *U* (rows) and *V* (columns), that is, the weight of the edge $(u_i,v_j)$ is *W*(*i*, *j*). The *k*-assignments, $1 \le k \le 4$, are: $M_1 = \{(u_2,v_1)\}$ of weight $w_1=10$, $M_2 = \{(u_2,v_1)$, $(u_1, v_2)\}$ of weight $w_2=10+8=18$, $M_3 = \{(u_2,v_1)$, $(u_1, v_2)$, $(u_3,v_3)\}$ of weight $w_3=10+8+5=23$ and $M_4 = \{(u_2,v_1)$, $(u_1, v_3)$, $(u_3,v_4)$, $(u_4, v_2)\}$ of total weight $w_4=10+5+4+4=23$. The sequence of weights (10, 18, 23, 23) is clearly concave and it forms the coefficients of the full characteristic maxpolynomial ${\bar{\chi }}_{A} (x) = x^4 \oplus 10x^3 \oplus 18x^2 \oplus 23x \oplus 23$.

## Computing the *k*-assignments

In Gassner and Klinz (G-K) (2010) introduced an efficient algorithm for a special kind of the parametric assignment problem. We present here (Sect. 5.1) the algorithm when applied in the max-plus setting to a matrix *W* with a parameter at all its entries. The result is the computation of the full characteristic maxpolynomial ${\bar{\chi }}_{W} (x)$. The coefficient of this maxpolynomial are the values of the corresponding *k*-assignments with a cost matrix *W*. Moreover, the G-K algorithm computes also the *k*-assignments themselves. However, these *k*-assignments refer to the essential terms of ${\bar{\chi }}_{A} (x)$. It is then easy to compute the *costs* of the remaining *k*-assignments. But what is special about the full characteristic maxpolynomial is that it is in full canonical form, so that the remaining *k*-assignments (the ones not computed by the G-K algorithm) refer to semi-essential terms. This property allows us to compute very efficiently, in addition to their costs, also the optimal *assignments* themselves, the ones on which these costs are attained (Sect. 5.2).

### Computing the *k*-assignments corresponding to essential terms

We present here the algorithm of Gassner and Klinz for the computation of the *k*-assignments that refer to the essential terms of the full characteristic maxpolynomial. The presentation is more similar to the one appearing in Hook (2016), which is clearer to understand in our opinion. The major steps of the algorithm are described in Algorithm 1.
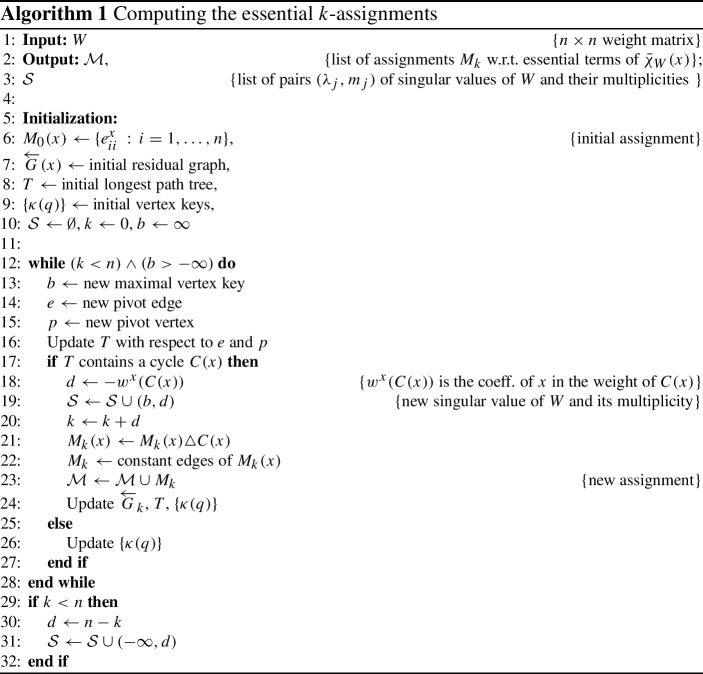


Given a weight (cost) matrix $W=(w_{ij})$ of size $n \times n$, we form the directed bipartite graph $K_{n,n} = G(U,V;\!\!E)$ enhanced with weights $w_{ij}$ applied to the “constant edges” $e_{ij}$ from the vertices $u_i$ to the vertices $v_j$. Then we add $n^2$ “parametric edges” $e^{x}_{ij}$ directed from $u_i$ to $v_j$, for $i,j=1,\ldots ,n$, where the weight of each parametric edge is set to be *x*. Finally, we add a root *r* and *n* edges $e^r_i$, each of weight 0, that start in *r* and terminate in $u_i$, $i=1,\ldots ,n$. These edges remain fixed throughout the algorithm, whereas the other edges may change direction, accompanied by negation of their weight. The resulting graph is denoted by *G*(*x*).

As in Hook’s presentation of the algorithm (Hook 2016), we begin with a value $b_0$ which is larger than the maximal entry of *W*. Clearly, for any value of $x \in [b_0,\infty )$, an assignment (matching of cardinality *n* of maximal weight) in *G*(*x*) contains *n* pairwise non-adjacent parametric edges, so that the weight of the assignment is $w_0 (x) = nx$. The weight of this assignment corresponds to the term $x^n$ of the full characteristic maxpolynomial ${\bar{\chi }}_{W} (x) = {{\,\mathrm{\mathrm {maxperm}}\,}}(x 0 \oplus W)$. Then the value of the parameter *x* is gradually decreased and the constant entries of *W* are exposed. This is done in a bounded number of discrete steps, where at each step a new value $b_i \le b_{i-1}$ is computed and several updates are performed. If during these updates some special condition is fulfilled (the emerging of a cycle after updating the spanning tree, see below) then $b_i$ is the value of the next max-plus singular value $\lambda _j$ and a new assignment is computed. The new assignment has less parametric edges than the previous one, and the difference is the multiplicity of $\lambda _j$ (although, in fact, the singular values $\lambda _j$ need not be distinct and the actual multiplicity of $\lambda _j$ is then the sum of the corresponding multiplicities). The weight $w_k (x) = a_k + (n-k)x$, , of the new assignment that applies to the current value of *x* corresponds to a term $a_k x^{n-k}$ of ${\bar{\chi }}_{W}(x)$, that is, $w_k = a_k$ is the total weight of a *k*-assignment in $G(U,V;\!\!E)$.

The question now is how do we compute the essential *k*-assignments themselves? The G-K algorithm proceeds as follows. First we let the initial assignment $M_0 (x)$ for $b_0 \le x < \infty$ to consist of the parametric edges $e^{x}_{ii}$, $i=1,\ldots ,n$. Then we construct the **residual graph** $\overleftarrow{G}(x)$. The residual graph is obtained from the graph *G*(*x*) by reversing the direction of the matching edges $e^{x}_{ii}$ and negating their weights. A main tool of the algorithm consists of constructing and maintaining in the residual graph a parametric longest path tree *T* according to the algorithm of Young et al. (1991) (in Young et al. (1991) the constructed tree solves the analogous **parametric shortest path** problem). This is a spanning tree with the property that the sum of weights of a path from the root to any vertex gives the longest existing path that reaches this vertex. At the beginning the tree *T* may consist e.g. of the edges $e^{r}_{i}$, for $i = 1,\ldots ,n$, $e^{x}_{1j}$, for $j=2,\ldots ,n$, and $e^{x}_{21}$, which gives a path of length (weight) 0 to each of the vertices $u_i$ and a path of length *x* to each of the vertices $v_j$.

We notice that the construction of a longest path tree *T* is only possible when $\overleftarrow{G}(x)$ does not contain (reachable) cycles of positive weight: once such a cycle exists then it can be taken unlimited number of times while increasing the weight of the path indefinitely. This property is crucial: when the value of *x* drops below some threshold $b_i$ then the graph *G*(*x*) contains a matching of *n* pairs of vertices whose total weight is larger than the current assignment (if such a matching does not exist the algorithm terminates). When $x < b_i$ the construction of a longest path tree is not possible anymore and this is demonstrated in the algorithm in the emergence of a cycle of positive weight when the tree is updated (see Ahuja et al. 1993 for this property in terms of network flow). At that point we get a new max-plus singular value $\lambda _j = b_i$.

In general, as the value of *x* decreases then *T* needs to be updated more often than updating the assignment. The update of *T* is performed by trying to replace an edge $e=p \rightarrow q$ by an edge $e'=p' \rightarrow q$ (a **pivot edge**). If the sub-tree of *T* rooted at *q* does not contain the vertex $p'$ then the replacement succeeds and the new tree is well-defined. After updating the vertex keys (as explained below) the algorithm continues by looking for the next pivot edge. Otherwise (when the sub-tree rooted at *q* contains $p'$), the replacement of *e* by $e'$ results in a cycle $C(x) = q \rightarrow q_1 \rightarrow \ldots \rightarrow q_{t-1} \rightarrow p' \rightarrow q$ of positive weight. In this case we do the following. The cycle *C*(*x*) is alternating between edges of the current assignment $M_k (x)$ and non-matching edges. We change the direction of the edges of *C*(*x*) and negate their weights (thus turning the cycle to be of negative weight) while obtaining a new residual graph $\overleftarrow{G}(x)$. If the current assignment is $M_k (x)$ then the new assignment is $M_{k+d} (x) = M_k (x) \triangle C(x)$, the symmetric difference of $M_k(x)$ and *C*(*x*). Here $k+d$ is the number of constant edges in the new assignment and $d=-w^x(C(x))>0$, where $w^x(C(x))$ denotes the integral coefficient of *x* in the total weight of the edges of *C*(*x*). *d* is also the multiplicity of the corresponding max-plus singular value. Finally, we update the longest path tree: the edges of the new tree go in the direction $q \rightarrow p' \rightarrow q_{t-1} \rightarrow \ldots \rightarrow q_1$.

It remains to show how to find the pivot edge $e'$ with which we update the longest path tree. To reach this goal (Gassner and Klinz 2010) apply the algorithm of Young et al. (1991). For each directed edge *e* of the residual graph $\overleftarrow{G}_k (x)$ let its weight be written as $w(e)=w^c(e)+w^x(e)x$, with  is the constant part and $w^x(e) \in \{0,-1,1\}$ is the parametric part. Then let $w(q)=w^c(q)+w^x(q)x$, where  and $w^x(q) \in [-n \, .. \, n]$, be the weight of a vertex *q* of $\overleftarrow{G}_k (x)$, defined as the sum of the weights along the path from the root to *q* in the current tree. For each directed edge $e = p \rightarrow q$ of the residual graph $\overleftarrow{G}$ its **key** is defined to be

5.1 $$\begin{aligned} \kappa (e) = \frac{(w^c(p)+w^c(e))-w^c(q)}{w^x(q)-(w^x(p)+w^x(e))}, \end{aligned}$$

where $\kappa (e)$ is defined to be $-\infty$ if the denominator in (5.1) is not positive. Note that $\kappa (e)$ gives the value of *x* at which the weight of the alternative path to *q* through *e* becomes equal to the weight of the path to *q* along the current tree, and for $x < \kappa (e)$ the alternative path is the longer one. The key of a vertex *q* is then defined to be the maximum over the keys of all edges that terminate in *q*:

5.2 $$\begin{aligned} \kappa (q) = \max _p \{\kappa (e) \, : \, e=p \rightarrow q\}. \end{aligned}$$

The update of the tree *T* is done by choosing a vertex *q* of $\overleftarrow{G}_k (x)$ of maximal key (a **pivot vertex**) as well as an edge $e=p \rightarrow q$ with $\kappa (e) = \kappa (q)$ (a pivot edge) and performing the update as described above. Then we need to update the keys of all the vertices in the sub-tree with root *q* before continuing.

For more details we refer to Gassner and Klinz (2010), Hook (2016) and Young et al. (1991).

### Computing the *k*-assignments corresponding to semi-essential terms

Algorithm 1 computes the *k*-assignments which correspond to all the essential terms of the full characteristic maxpolynomial ${\bar{\chi }}_{W}(x)$ and probably some which correspond to semi-essential terms. Since ${\bar{\chi }}_{W}(x)$ is in FCF, we can easily deduce the values of the missing *k*-assignments. The question is how to compute the missing *k*-assignments themselves. The computation of all the *k*-assignments already in Algorithm 1 succeeds when each newly computed max-plus singular value $\lambda _j$ comes with multiplicity 1 (we remind that this is not necessarily its actual multiplicity since the computed singular values are not necessarily distinct). The G-K algorithm does not address the case of semi-essential terms, and it can be shown that in the presence of multiple pivot edges at the same time, the choices we make influence the multiplicities of the newly computed singular values. It is not clear whether it is always possible to choose the pivot edges in Algorithm 1 in such a way that the singular values have multiplicity 1, and if yes - if it can be done in an efficient way. Nor isn’t it clear whether it is possible to direct the algorithm in such a way that the max-plus singular values will occur in full multiplicity, that is, that each computed new singular value will be different from the previous one, although one can try to choose the pivot edges in a way that serves this or the other purpose.

However, we will show that after obtaining the *k*-assignments from Algorithm 1, the remaining *k*-assignments, those that correspond to semi-essential terms of ${\bar{\chi }}_{W}(x)$, are easy to compute.

#### Theorem 5.1

Let ${\mathcal {M}}$ be the set of *k*-assignments computed by Algorithm 1 when given an *n*-agents, *n*-tasks assignment problem. Let $I \subseteq [n]$ be the set of integers *k*, for which ${\mathcal {M}}$ contains a *k*-assignment. Then it is possible to compute efficiently out of ${\mathcal {M}}$ the set of all the remaining *k*-assignments, for $k \in [n] {\setminus } I$.

#### Proof

We go over the sequence of *k*-assignments in ${\mathcal {M}}$ until we find a gap in the computed assignments. Then we compute the missing assignments, find the next gap and compute the next missing assignments and so on.

So, suppose that ${\mathcal {M}}$ contains an *l*-assignment $M_l$ and the next computed assignment is an $(l+d)$-assignment $M_{l+d}$, where $d>1$. We know that because the assignments refer to essential terms of the corresponding full characteristic maxpolynomial, which is in full canonical form, then the missing assignments refer to semi-essential terms. This means that the weights $w(M_{l+i})$ of the missing assignments $M_{l+i}$, for $i=1,\ldots ,d-1$, are evenly spaced. Let

$$\begin{aligned} h_i = w(M_{l+i}) - w(M_{l+i-1}), \qquad \text{ for } i=1,\ldots ,d, \end{aligned}$$

be the weight difference between successive *k*-assignments, and let

$$\begin{aligned} h = w(M_{l+d}) - w(M_l) \end{aligned}$$

be the weight difference between the computed assignments $M_l$ and $M_{l+d}$. By the above,

$$\begin{aligned} h_i = \frac{h}{d}, \qquad \text{ for } i=1,\ldots ,d. \end{aligned}$$

As in the Proof of Proposition 4.2, in order to compute the missing assignments, we start with the assignment $M_l$ and transform it to the assignment $M_{l+d}$ in *d* steps. The set of matched pairs in $M_l \cup M_{l+d}$ form (maximal) disjoint paths of four possible types.

1. Edges $$(u_i,v_j) \in M_l \cap M_{l+d}$$;
2. Maximal alternating paths of even lengths;
3. Maximal augmenting paths of $M_{l+d}$ with respect to $M_l$;
4. Maximal augmenting paths of $M_l$ with respect to $M_{l+d}$.

The paths of type  and contain the same number of matches of $M_l$ and of $M_{l+d}$. Each path of type  contains one more match of $M_{l+d}$ than of $M_l$ and the other way round for paths of type . Since $M_{l+d}$ contains *d* more matches than $M_l$, the number of paths of type  is greater by *d* than the number of paths of type .

For each alternating path *p* of type  we have, by the maximality of the assignments $M_l$ and $M_{l+d}$, the following equality of weights:

$$\begin{aligned} w(p \cap M_l) = w(p \cap M_{l+d}). \end{aligned}$$

Suppose that there exists at least one path of type . Then for each pair $(p_3,p_4)$, where $p_3$ is a path of type  and $p_4$ is a path of type , we have, by maximality of $M_l$ and $M_{l+d}$,

$$\begin{aligned} w((p_3 \cup p_4) \cap M_l) = w((p_3 \cup p_4) \cap M_{l+d}). \end{aligned}$$

Equivalently, there is a constant *c*, such that for every augmented path of type  or of type ,

5.3 $$\begin{aligned} w(p^{odd}) - w(p^{even}) = c, \end{aligned}$$

where $p^{odd}$ refers to the edges of *p* in the odd places and $p^{even}$ refers to the edges of *p* in the even places.

Thus, without changing its total weight, we can form a (possibly new) assignment $M_{l+d}$ out of the matched pairs of the given assignment $M_{l+d}$ of any *d* augmenting paths $p_1,\ldots ,p_d$ of type , and then add all the matched pairs of $M_l$ which are not in $p_1,\ldots ,p_d$. For each of these paths $p_i$ of type , let its “gain” be

$$\begin{aligned} g_i = w(p_i \cap M_{l+d}) - w(p_i \cap M_l). \end{aligned}$$

In case there is at least one path of type , we have seen in (5.3) that the $g_i$ are equal to each other. Otherwise, suppose, without loss of generality, that

5.4 $$\begin{aligned} g_1 \ge g_2 \ge \cdots \ge g_d. \end{aligned}$$

We will show that due to our special case also in case there are no paths of type  the $g_i$ are all the same. Let us construct the matchings ${\tilde{M}}_{l+i}$ as

$$\begin{aligned} {\tilde{M}}_{l+i} = {\tilde{M}}_{l+i-1} \triangle p_i, \qquad \text{ for } i=1,\ldots ,d\!-\!1, \end{aligned}$$

where ${\tilde{M}}_l$ is defined to be $M_l$. (At this point we do not know yet whether these matchings are (optimal) *k*-assignments.) Then we have,

$$\begin{aligned} g_i = w({\tilde{M}}_{l+i}) - w({\tilde{M}}_{l+i-1}), \qquad \text{ for } i=1,\ldots ,d, \end{aligned}$$

(with ${\tilde{M}}_l=M_l$ and ${\tilde{M}}_{l+d}=M_{l+d}$) and

5.5 $$\begin{aligned} \sum _{i=1}^{d} g_i = \sum _{i=1}^{d} h_i = w(M_{l+d}) - w(M_l) = h. \end{aligned}$$

By the maximality of the $(l+1)$-assignment,

5.6 $$\begin{aligned} g_1 \le h_1 = \frac{h}{d}, \end{aligned}$$

and it follows from (5.4), (5.5) and (5.6) that

$$\begin{aligned} g_1 = g_2 = \cdots = g_d = \frac{h}{d}, \end{aligned}$$

and thus each of the matchings ${\tilde{M}}_{l+i}$ is indeed an $(l+i)$-assignment, for $i=1,\ldots ,d~-~1$. $\square$


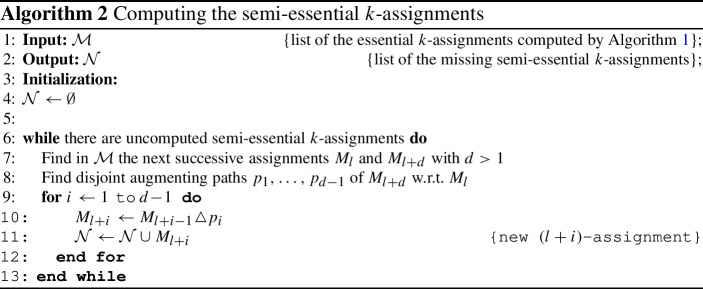


### Time complexity

The G-K algorithm computes the *k*-assignments that refer to essential terms and possibly some of the semi-essential terms in time ${\mathcal {O}}(n^3)$.

In Algorithm 2 we compute the semi-essential *k*-assignments that remain to be computed after completing the G-K algorithm, thus obtaining *all* the *k*-assignments, for $k=1,\ldots ,n$. Our aim here is to show that the running time of Algorithm 2 is ${\mathcal {O}}(n^2)$. The cause for being able to achieve such an improved running time, where, in general, solving a single *k*-assignment is of time complexity ${\mathcal {O}}(n^3)$ with the known algorithms, is that, as we showed in Sect. 5.2, the remaining semi-essential assignments can be extracted out of the already computed assignments with the G-K algorithm without the need to refer to the original data of $n^2$ agent-task costs.

Given a list of the *k*-assignments computed by the G-K algorithm, our mission is to fill-in the gaps in this list. We can assume that the *k*-assignments are in the following data structure. For each vertex $u_i$ there exists an array $U_i$ of size *n*, so that $U_i[k]=j$ in case $u_i$ and $v_j$ are matched in the *k*-assignment, and similarly for every $v_j$ corresponds an array $V_j$, where in this case $V_j[k]=i$ for the same match in the *k*-assignment.

So, suppose that the assignments $M_l$ and $M_{l+d}$, $d>1$, are given by the G-K algorithm, whereas the in-between *k*-assignments are missing. Starting at any matched pair of $M_{l+d}$, we can extend it to a maximal alternating path by following the pointers at $U_i[l]$, $U_i[l+d]$, $V_j[l]$ and $V_j[l+d]$, for $i,j \in \{1,\ldots ,n\}$. That is, if $U_i[l+d]=j$ (a $(u_i , v_j)$ match in $M_{l+d}$), then we look at $V_j[l]$. If $V_j[l]=k$ (a $(v_j , u_k)$ match in $M_l$) then we look at $U_k[l+d]$, and so on. The total number of matches (or edges) in $M_l$ and in $M_{l+d}$ together is at most 2*n*, so that the process of finding all the alternating paths is done in time ${\mathcal {O}}(n)$. There are at least *d* augmenting paths of type , that is, (maximal) paths that start and end at matches of $M_{l+d}$. Hence, in general, we need not find all the alternating paths, but stop once we find $d-1$ paths, say, $p_1,\ldots ,p_{d-1}$, of type .

Next, we compute the assignments $M_{l+1}, \ldots ,M_{l+d-1}$ as follows. Suppose that we already computed $M_{l+1}, \ldots ,M_{l+i-1}$. Then we form $M_{l+i}$ by taking $M_{l+i-1}$ and replacing the matches in $p_i$ that belong to $M_l$ (and to $M_{l+i-1}$) by the matches in $p_i$ that belong to $M_{l+d}$ (that is, obtaining the symmetric difference of $M_{l+i-1}$ and $p_{i}$), thus increasing the overall number of matches by one. This can be done in time ${\mathcal {O}}(n)$, so that the overall time for obtaining $M_{l+i}$ is ${\mathcal {O}}(n)$.

Since the number of *k*-assignments to be computed by Algorithm 2 is less than *n*, we achieve an ${\mathcal {O}}(n^2)$ time complexity for computing all the missing *k*-assignments.

We conclude that the time complexity for computing all the *k*-assignments, $k=1,\ldots ,n$, in the combined G-K Algorithm and Algorithm 2 is ${\mathcal {O}}(n^3)$.

In special cases, where there are many assignments that are left to be computed after completing the G-K algorithm, the fact that these extra assignments are computed more efficiently may reduce the overall running time. For example, such a case may occur when many of the $n^2$ agent-task costs are equal to each other.

## Data Availability

Data sharing not applicable to this article as no datasets were generated or analysed during the current study.
